# Effectiveness of a new multi-component smoking cessation service package for patients with hypertension and diabetes in northern Thailand: a randomized controlled trial (ESCAPE study)

**DOI:** 10.1186/s13011-019-0197-2

**Published:** 2019-02-22

**Authors:** Myo Nyein Aung, Motoyuki Yuasa, Saiyud Moolphate, Thaworn Lorga, Hirohide Yokokawa, Hiroshi Fukuda, Tsutomu Kitajima, Susumu Tanimura, Yoshimune Hiratsuka, Koichi Ono, Payom Thinuan, Kazuo Minematsu, Jitladda Deerojanawong, Yaoyanee Suya, Eiji Marui

**Affiliations:** 1 Advanced Health Science Institute, and Faculty of International Liberal Arts, Juntendo University, Hongo 2-1-1, Bunkyo-ku, Tokyo, 113-8421 Japan; 2 WHO Collaborating Center for Medical Education, Faculty of Medicine, Chulalogkorn University, 5th fl Ananda Mahidol Building, 1873 Heneri Dunant road, Pathuwam, Bangkok, 10330 Thailand; 3 Faculty of International Liberal Arts and Department of Public Health, School of Medicine, Juntendo University , Tokyo, Japan; 4 Department of Public Health, Faculty of Science and Technology, Chiang Mai Rajabhat University, Chiang Mai, Thailand; 5 Boromrajonani College of Nursing, Lampang, Thailand; 6 Department of General Medicine, Juntendo University School of Medicine, Tokyo, Japan; 7 Faculty of Social Science, Kyorin University, Tokyo, Japan; 8 Department of Public Health Nursing, Mie University Graduate School of Medicine, Tsu, Japan; 9 Department of Opthalmology, Juntendo University School of Medicine, Tokyo, Japan; 10 Graduate School of Education, Nagasaki University, Nagasaki, Japan; 11 Maetha Hospital, Lampang, Thailand; 12 Department of Human Arts and Sciences, University of Human Arts and Sciences, Saitama, Japan

**Keywords:** Tobacco, Family, Smokerlyzer, Diary, NRT, Coaching, Hand-rolled cigarette

## Abstract

**Background:**

Smoking cessation is an achievable behavioral change, which reduces the risks of cardiovascular diseases, cancers and tobacco-related diseases. There is a need for an effective smoking cessation service for low and middle income country settings where the smoking rate is generally very high whilst a cessation service is not usually accessible. This study devised a new smoking cessation service package and assessed its effectiveness in the primary health care setting of northern Thailand.

**Methods:**

This randomized controlled trial was centered at Maetha district hospital, Lampang province, Thailand, and its network of mobile non-communicable disease clinics at seven primary care units. A total of 319 eligible patients who consented to participate in the study, were randomly allocated to an intervention arm (160) and a control arm (159), applying block randomization. The multi-component intervention service consisted of:

1. regular patient motivation by the same nurse over a 3-month period;
2. a monthly piCO+ Smokerlyzer test for 3 months;
3. continual assistance from a trained family member, using a smoking-cessation- diary; and
4. optional nicotine replacement chewing gum therapy.

The control group received the routine service comprising of brief counseling and casual follow-up. Smoking cessation, confirmed by six months of abstinence and the piCo+ Smokerlyzer breath test, was compared between the two services after a year follow-up.

The trial is registered as an international current control trial at the ISRCTN registry. ISRCTN89315117.

**Results:**

The median age of the participants was 64 years, with females constituting 28.84%. Most of the participants smoke hand-rolled cigarettes (85%). The intervention arm participants achieved a significantly higher smoking cessation rate than the control arm 25.62% vs 11.32%, with an adjusted odd ratio of 2.95 and 95% confidence interval 1.55–5.61.

**Conclusion:**

In relation to accessing smoking cessation services within the primary health care setting, participants who received the evidence-based intervention package were about three times more likely to succeed in giving up smoking than those who received the routine service. Utilizing community resources as major intervention components, the evidence from this trial may provide a useful and scalable smoking cessation intervention for low and middle income countries.

**Trial registration:**

Current controlled trials ISRCTN89315117.

WHO international clinical trial identifier number: U1111–1145-6916; 3/2013.

## Background

Smoking cessation is a global priority representing one of the most needed public health interventions in order to prevent millions of deaths and morbidity [1]. However, it is not a routinely accessible service in many low and middle income developing countries (LMIC) such as Thailand [2]. Despite scientific evidence indicating the benefits of smoking cessation methods, including brief advice, nurse counseling, motivational interviewing, and pharmacological treatment, such as nicotine replacement therapy (NRT), a number of social, cultural and health system factors challenge the integration of a smoking cessation service into primary health care settings [1].

The smoking rate in Southeast Asian countries such as Thailand, Myanmar, Cambodia, Lao and Vietnam are very high, ranging from 20 to 40% [3]. A randomized controlled trial which tests the practical effectiveness of a new evidence-based smoking cessation service versus the traditional routine service is urgently required for effective tobacco control and relieving the non-communicable diseases (NCD) burden in Southeast Asia.

Smoking is a risk behavior influenced by social and cultural factors in addition to individual addiction to tobacco and nicotine. Some of the existing smoking cessation techniques that have proved to be effective in developed countries may, however, be unsuitable for LMICs. Nicotine patches, for example, may not be user-friendly in humid, tropical weather [4]; lengthy motivational interviews are not practical due to the inadequate number of health professionals [5]; and pharmaceutical prescriptions are less affordable in the absence of insurance schemes reimbursing their use. Social support techniques, therefore, such as family support and buddy-support, seem more feasible in terms of the availability of community resources.

Whilst the existing literature does not provide conclusive evidence in favor of family support intervention, positive associations have been made in cross-sectional and cohort studies between smokers living with their family and their successful smoking cessation [6–8]. An intervention that engages family support to assist the smokers’ attempts to give up smoking, involving cessation remedies that are tailored to the needs of the individual, may represent an interesting and practicable smoking cessation service for Southeast Asian LMICs.

**Fig. 1:**
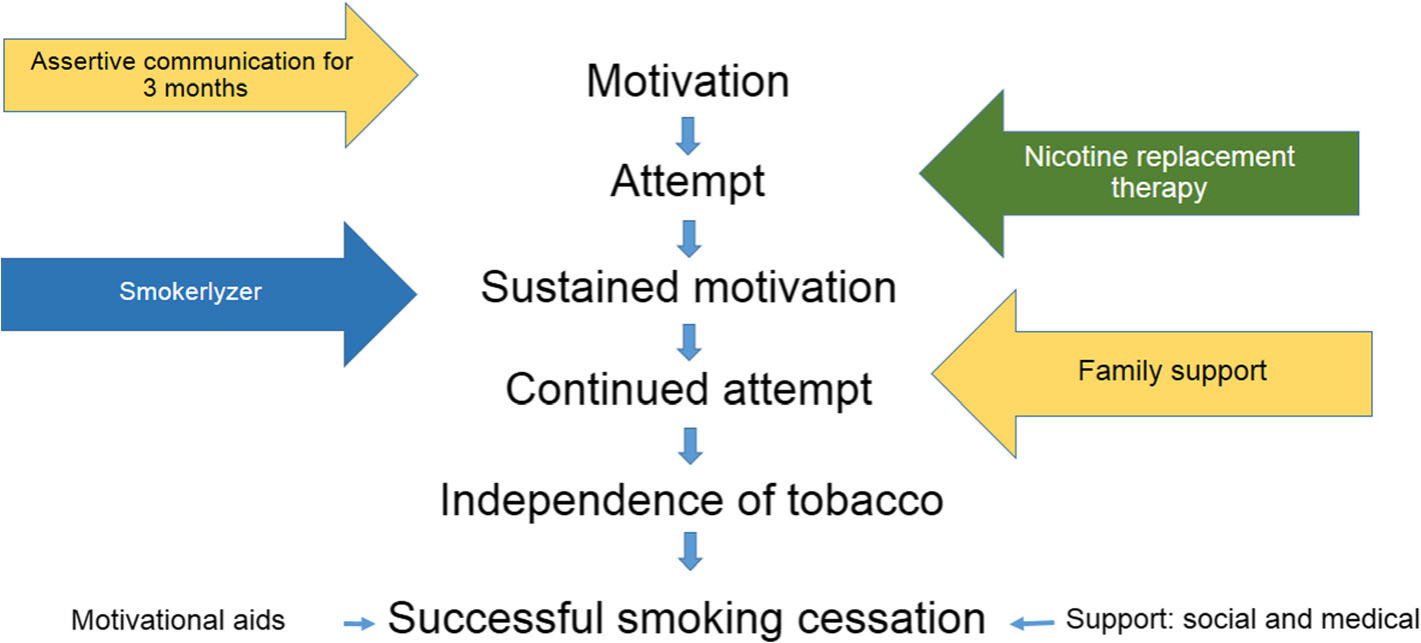
Conceptual model of newly-devised smoking cessation service

In this study, a multiple-component smoking cessation intervention (Fig. 1) was carefully designed and integrated within primary health care clinics, and compared to the routinely available service in a randomized controlled trial. The objectives of the trial were: (1) to compare levels of smoking cessation between the intervention new service package arm and the control routine service arm over a six-month period; and (2) to compare the Smokerlyzer-confirmed smoking cessation rates between the intervention new service package arm and the control routine service arm.

## Method

### Study design and patient eligibility

A two parallel group randomized controlled trial was conducted**-**the details of the methods applied in this trial are published in a previous study [9]). The acronym ESCAPE, used in the present trial, refers to the evidence-based augmented package for smoking cessation [10].

The study was implemented in Thailand from June, 2012 to December, 2015. The trial was centered at Maetha Hospital, Lampang province, northern Thailand. Recruitment for the study started simultaneously at seven primary health care units within the mobile NCD clinic network of Maetha district, Lampang province, in June 2012. The setting of the trial was that of rural districts where people often grow tobacco in their gardens and consume home-made hand-rolled cigarettes.

Eligibility criteria were applied to enrol individual patients into the study.

The inclusion criteria were:

1. Current smoker with diabetes
2. Current smoker with hypertension
3. Current smoker with both diabetes and hypertension
4. A smoker that has never succeeded in giving up smoking
5. Male or female
6. Age range from 35 to 80 years

The exclusion criteria were:

1. Any female patient who is pregnant or planning to become pregnant
2. Patient aged younger than 35 years
3. Patient with documented type I diabetes
4. Patient with cancer
5. Patient with severe chronic pulmonary diseases using home oxygen therapy
6. Patient with known diagnosis of a previous cardiovascular disease (CVD) event

### Randomization and masking

**Fig. 2:**
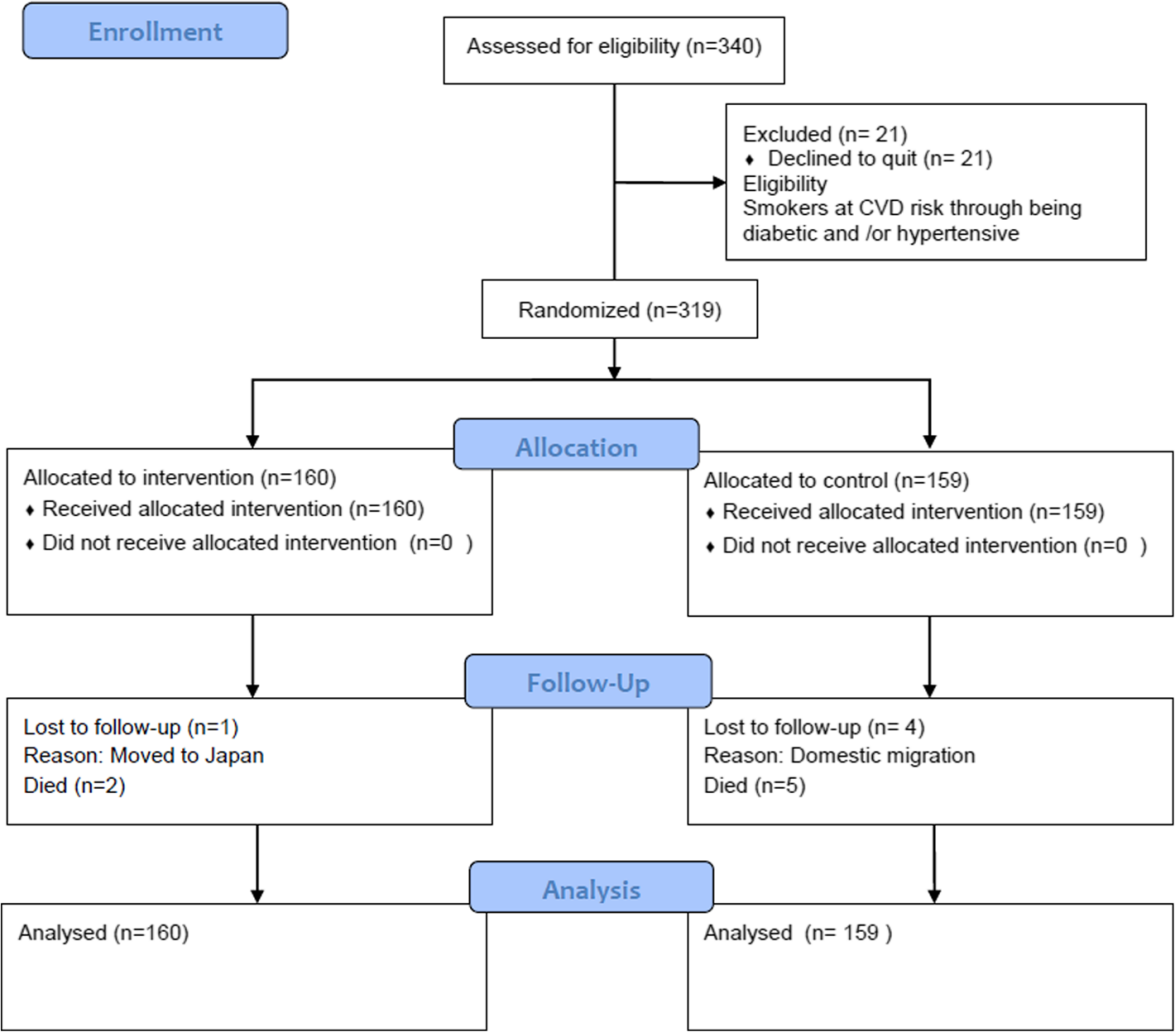
Consort flow chart for enrollment and follow-up plan for randomized controlled trial

A total of 319 eligible participants were randomized (Fig. 2). The sites of randomization were seven primary health care units. Randomization was carried out in blocks of 32. The nature of the present study did not allow for blinding, however, the allocation of participants was concealed to the research staff until the opaque envelope containing the pre-generated random sequence for allocation was opened. The possibility of residual investigators’ bias, therefore, was controlled. The components in the intervention new service package and the routine smoking cessation service were so different that the chance of contamination was considered minimal.

### Procedures for the intervention and control arms

#### Intervention arm

The smoking cessation service package for the intervention arm consisted of the following elements: [10]

**Fig. 3:**
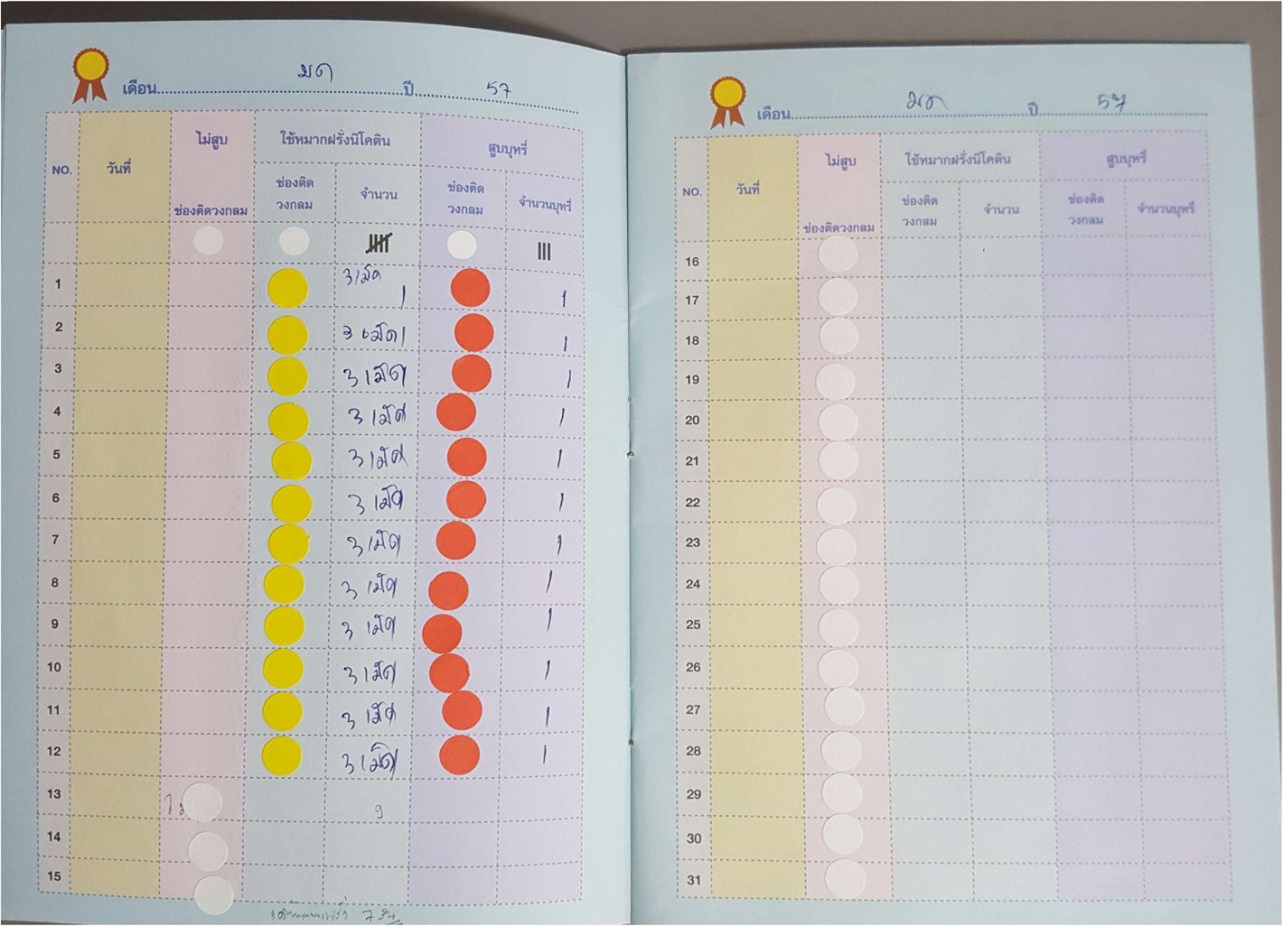
"Family-assisted smoking cessation diary" applied to engage family assistance in smoking cessation attempt. Note: The red colored sticker marked the days of tobacco smoking: The yellow colored stickers marked the use of nicotine replacement therapy and the white colored stickers maked no-smoking days

1. Assertive communication was carried out between the nurse and the patient to achieve the goal of smoking cessation, as opposed to the conventional patient counseling undertaken at the first meeting between the smoking patient and the primary care unit (PCU) nurse. This aimed to encourage the patient to attempt to stop smoking by providing a clear explanation relating to smoking cessation. This communication was sustained by the same nurse repeating the advice each month for the 3 months following enrolment.
2. A piCO+ Smokerlyzer was used to show the level of carbon monoxide (CO) the patient breathed out and the improvement of the patient’s lung health over three successive months. As a result, participants in the new service arm group could see both the level of their nicotine dependence, via the colored diode light in the Smokerlyzer breath analysis, and also the level of CO in their expired air. They could see the real result of their attempts to give up smoking in terms of a declining ppmCO result and the changing diode light from red to yellow to green, with green as the target light. It was considered that these achievable and visible results could serve as a motivational aid.
3. At the enrolment of the participant, a member of the participant’s family was assigned and trained by the PCU nurse to monitor, remind and assist the smoker until smoking cessation could be achieved. An attractive “family-assisted smoking cessation diary” was given to the family member along with three different colored stickers to record the participant’s choice of smoking each day: smoking (red), NRT chewing gum (blue or yellow), and non-smoking (white) (Fig. 3). This diary was expected to motivate the smoker and the assisting family member by serving as a reminder to them both.
4. Patients, who were willing, were given NRT in the form of nicotine chewing gum when they experienced nicotine withdrawal symptoms [11, 12].

#### Control arm

The control group participants received the existing routine service for smoking cessation. The routine health service smoking cessation package comprised the following elements:

1. At the first meeting with the smoking patient, the hospital healthcare worker advised the patient to stop smoking on medical grounds, and explained how smoking cessation could be successful.
2. The patient was asked questions about their smoking habit in order to measure their level of nicotine dependency.
3. The patient was reminded by the healthcare worker on subsequent visits to the hospital to continue trying to give up smoking.
4. The patient was requested by the healthcare worker to inform if and when their smoking cessation had been achieved.

#### Training and preparation

Nurses from seven primary health care centers were trained to deliver the intervention service package. Pre-intervention training workshops, carried out two times, explained how to provide assertive communication in the first three months, how to apply the smoking cessation diary to motivate the smoker and family member, how to provide nicotine replacement therapy, and how to use a Smokerlyzer. A pilot practice of the new service was carried out before the trial launch.

#### Study outcomes

Each participant was followed for one year after enrolment into the study. Smoking cessation was assessed at six months and twelve months.

**The primary outcome** of the study was of smoking cessation for six months at the end of the one year follow-up. This was measured via the smoker’s self-reporting of smoking cessation over the previous 24 h, self-reporting of smoking cessation over the previous seven days, and a confirmatory measurement of carbon monoxide in parts per million (ppmCO) using a piCo^+^Smokerlyzer [13]. We applied this chemical analysis of breath air in order to avoid possible errors in self-reported cessation. These measurements are longitudinally conducted at base-line, 3 month follow up and 6 month follow up to confirm behavior change. At each follow up, the participants undergo all the outcome measurement. When someone cannot stop continuously from zero to sixth months of follow up, this cases was not considered as smoking cessation.

**Baseline measurements** included social and demographic information, the nicotine dependence level (assessed using the Fagerstrom Nicotine Dependence Test (FTND)) [11, 12, 14], clinical information including the presence of hypertension, blood pressure, or diabetes, the blood glucose level, waist circumference, body mass index (BMI), health literacy for cardiovascular diseases, and CVD risk assessment.

**Participants’ motivation** to give up smoking was assessed by the validated Motivation To Stop Scale (MTSS) [15], whilst **family support** was measured by applying a newly-developed and validated four-point, 16-item instrument designed to measure the level of family assistance in smoking cessation.

### Statistical analysis

The sample size was calculated to a power of 90%, based on the year 2012 smoking cessation rate at Maetha hospital (9%), and the estimated cessation rate of the evidence-based package (24%). There was a 95% confidence interval with 20% of the calculated sample added on to compensate for the loss of follow-up. STATA version 11 SE was used to analyze the data. Analysis of baseline characteristics was applied via a chi-squared test and the Wilcoxon signed-rank test. Tobacco exposure was compared between the two study arms by comparing the pack-years, FTND scores, and Smokerlyzer ppmCO levels within a MANOVA model. Primary analysis involved an intention-to-treat (ITT) approach. Smoking cessation rates between the two study arms were compared applying a chi-squared test. In addition, the association between the intervention and smoking cessation for six months was analyzed via multiple logistic regression analysis. The final model of multiple logistic regression analysis adjusted nicotine dependency as FTND scores categories (< 5 and ≥ 5) and tested effect modification applying interaction command. Strength of association between intervention and outcome was shown in adjusted odds ratio (aOR) with 95% confidence interval (95%CI).

### Ethic approval and trial registration

The study was approved by the Juntendo University Ethical Committee, Japan, with approval number 2012194, and the Institutional Review Board at Boromarajonani College of Nursing, Lampang, Thailand, with approval number E2556/005. It was registered as an international current control trial at the ISRCTN registry. The ISRCTN registration number ISRCTN89315117 was assigned on 9th July 2013 [10]. Written consent was obtained from each of the participants after a thorough explanation was given about what would happen to them during the study, as well as their autonomous decision to give up smoking.

## Results

A total of 319 participants were randomized and included in the intention-to-treat analysis with 160 (50.3%) in the intervention arm and 159 (49.7%) in the control arm. The median age of the study group was 64 years, with females constituting 28.84%. The median duration of smoking was 46 years, and the median pack-year was 9.2. Hand-rolled cigarettes were the most common type of tobacco consumed in both study arms [16]. Participants were at cardiovascular disease (CVD) risk through being either hypertensive or diabetic, or both.

At the baseline, social, demographic, and clinical characteristics of the participants were balanced between the intervention arm and the control arm. Tobacco exposure, in terms of the age the smoker started smoking and the duration of smoking, were not significantly different. In addition, within the MANOVA model, the pack-year, the level of exhaled carbon monoxide level, and the Fagerstrom scores did not differ significantly between the two study arms. The smokers’ motivation for smoking cessation, measured using the MTSS scale, as well as the level of family support, also did not differ significantly between the two study arms. Only 36.88% of participants in the intervention group used NRT.

At the six-month follow-up, the smoking cessation rate was significantly higher in the intervention group compared to the control group. At the twelve-month follow-up, the smoking cessation rate improved in both study arms but the smoking cessation rate in the intervention group was statistically significantly higher than in the control group (Table 1). At the twelve- month follow-up, 41 participants (25.62%) in the intervention group, and 18 (11.32%) in the control group achieved the primary outcome, with smoking cessation for 6 months confirmed via measurement of the CO ppm level in the expired breath using a Smokerlyzer, in addition to the self-reporting for 6 months of smoking cessation over the previous 24 h, and over the previous 7 days. The result of intention-to-treat analyses was statistically significant. The power of the trial was 89% for the effect size in the primary outcome result.

**Table 1:** Baseline characteristics of the participants

Baseline characteristics	Intervention Arm (160)		Control Arm (159)		*P*
Social and demographic information	*n*	%	*N*	%	
Age, Median (IQR)	64 (56–72)		64(55–73)		ns^>^
Gender					ns^+^
Female	46	28.93	46	28.75	
Clinical information					ns^+^
Hypertension	115	72.33	105	47.73	
Diabetes	25	15.72	35	21.88	
Both	15	9.43	17	10.63	
Tobacco exposure					ns*
Age started smoking (years, Median (IQR))	16 (13–20)		18 (15–18)		ns^>^
Duration of smoking (years, Median (IQR))	47 (47–56)		45 (45–55)		ns*
Pack-year Median (IQR)	10.31(6.15–17.13)		8.55 (5.85–13.75)		ns*
Fagerstrom score, Median (IQR)	2(1–3)		1(0–3)		ns*
Exhaled CO in ppm, Median (IQR)	8.5 (3–12)		6 (3–12)		ns*
Types of smoking					ns
Cigarette	18	11.25	16	10.06	
Cheroots	4	2.50	3	1.89	
Hand-rolled	137	85.63	134	84.28	
Other factors related to smoking cessation					
Motivation to stop smoking, Median (IQR)	4 (3–5)		3 (3–5)		ns^>^
Family support at entry, Median (IQR)	42 (35–43)		40(35–43)		ns^>^
Five-itemed health literacy assessment, Median (IQR)	19 (17–20)		19(16–20)		ns^>^

Note: 5 participants in intervention arm and 2 participants in control arm are known diagnosis of hypertension but with normal blood pressure at the time of recruitment and follow up

*Multivariate analysis of variance (MANOVA): *IQR* interquartile range: ^+^chi-squared test: ^>^Wilcoxon signed-rank test: *ns P* > 0.05 and statistically not significant

Multiple logistic regression analysis was applied to test the association of the intervention to the study outcome. Statistical model tested possible effect modification of the nicotine dependency (FTND) scores. The intervention was found to be a significant factor associated with successful smoking cessation with an adjusted odds ratio (aOR) 2.95 and 95% confidence interval (CI) 1.55–5.61. Patient’s nicotine dependency level did not influence the outcome significantly (Table 2).

**Table 2:** Multivariate analysis of smoking cessation interventions and outcome

	OR	95% CI	aOR	95% CI	*P*
Intervention service package	2.70	1.47–4.94	2.95	1.55–5.61	0.001
High nicotine dependency Fagerstrom score ≥ 5	0.31	0.11–0.88	0.20	0.45–0.90	0.36

*OR* Odds ratio from univariate analysis, *95% CI* 95% Confidence Interval, *aOR* adjusted Odds ratio from multivariate logistic regression analysis

Four participants in the intervention arm and one participant in the control arm were lost to follow-up (1.57%). The number of deaths of participants in the intervention arm was two (1.25%), with five deaths (3.24%) in the control arm. The causes of death were ascertained as non-trial related.

## Discussion

WHO recommendations state that strengthening the health system for the prevention of NCD requires trained professionals assigned to a smoking cessation service, as well as diagnostics and equipment for the measurement of nicotine dependence, medication to treat nicotine withdrawal, and universal coverage of smoking cessation services [17]. When smokers are given the opportunity to stop smoking within a systematic approach, they are able to choose to try to give up smoking. To what extent such opportunities are given to people in developing countries, however, is still in question [18]. In this study, the opportunity to stop smoking, via a well-designed service package of smoking cessation support, was given to smokers in a randomized controlled trial conducted in the community setting of Thailand, a middle-income developing country.

Smoking cessation is a very difficult behavior change to bring about, requiring determination on the part of the smoker, as well as physical, mental and social support to overcome tough periods of withdrawal symptoms (Fig. 1). Individuals requiring a combination of these factors may resist single method cessation intervention [19]. Therefore, in the present ESCAPE study, a smoking cessation service package was developed, assembling feasible, evidence-based intervention elements, and integrated into the primary health care service. This intervention comprised strategic communication to trigger, enhance and sustain smokers’ motivation to give up smoking, the provision of nicotine chewing gum therapy as a cessation aid to those needing and willing to use it, the continuous monitoring and feedback provided by use of a Smokerlyzer, and the sustained support for the smoker given by a family member (Figs. 1 and 3). The verified six-month smoking cessation rate was significantly higher amongst those receiving the intervention service package compared to those receiving the routine service (Table 3).

**Table 3:** Smoking cessation rates in the intervention and control arms

Intention-to-treat analysis	Intervention		Control		*P*	NNT
	*n*	%	*n*	%		
*N* = 319	160	50	159	50		
Smoking cessation at 6th month	50	31.25	22	13.84	< 0.001	5.7
Smoking cessation at 12th month	62	38.75	23	14.47	< 0.001	4.1
Cessation for 6 months, PPM confirmed	41	25.62	18	11.32	< 0.001	7

*PPM* Smokerlyzer COppm*, P P*-value in chi-squared test*, NNT* number need to treat

Although tobacco-smoking represents a global health challenge, smoking cessation services are not commonly available and accessible in many low and middle income countries [2]. When introducing a smoking cessation service to the primary health care setting of a developing country, the application of community resources should ensure that the intervention service is sustainable [20]. In this trial, family support was engaged to assist the smokers in their attempts to stop smoking. Socially-bonded and residing in the same house, family members are the most accessible human resource who can provide sustained care and assistance to the smoker in their attempts to give up smoking. A simple diary was used to motivate both the family member and the smoker. Every day the family member recorded the smoking choice the smoker made for a period of 1 year: smoking, chewing nicotine replacement chewing gum, or non-smoking (Fig. 3). This record itself might have served to reinforce the smoker’s motivation to succeed in giving up smoking. Smokers who live alone, or who do not have the support of a family member, may require assistance from other community resources such as health volunteers. Furthermore, for those people who do not have a strong social bond with their family members, this intervention may be weak.

The successful smoking cessation rate of 25% amongst the intervention group in the present ESCAPE trial was higher than the rates reported in recent trials conducted in the pragmatic settings of developing countries such as Syria, India and Pakistan [4, 21, 22]. Those studies applied two components: behavioral support and smoking cessation aids, and delivered the service in an integrated approach either at a disease-specific clinic, such as a diabetes or TB clinic, or at a primary health care center. Their results suggest that combined method interventions are more effective than single component interventions [4, 21, 22]. Cochrane’s updated review reported that when combining behavioral support and pharmacological treatment, smoking cessation intervention becomes more effective [19]. The uniqueness of the present trial was that the smokers were offered a multi-component intervention in which, for example, nicotine replacement therapy (NRT) chewing gum was provided as a tailored need to the smokers who indicated a need and willingness to use it. Pharmacological treatment was complementary, with only a third of the intervention participants using NRT chewing gum. The major compulsory components of the intervention package were the initial and continual communication between the nurse and smoker, the more frequent monitoring by use of a Smokerlyzer, and the sustained assistance provided to the smoker by their family member.

The strength of the current trial included the use of primary outcome measurements carried out via the scientific measurement of the smokers’ expired air, as well as via the smokers’ self-reporting of their smoking abstinence, completed over a period of 6 months. A year-long follow-up involving the smokers’ routine NCD clinic attendance, ensured a very low loss of follow-up and outcome achievement [23]. To minimize the chance of variation amongst the seven sites carrying out the intervention, pre-intervention training and standardization of protocol were undertaken. Both intervention and routine arm services were standardized and piloted. At the outset of the trial, the level of tobacco exposure amongst participants in the intervention arm was slightly higher (although it was statistically insignificant) than in the control group. Multivariate analysis, therefore, adjusted the FTND score in the final model. Eventually, the impact of intervention was not cofounded by the nicotine dependence.

The current study may have limitations. The relaying of information between participants in different study arms may occur in trials such as this one. This was likely to be minimal in the present study, however, considering a number of differences between the intervention and control arms, such as the provision of NRT, the application of smoking cessation diaries, and the use of Smokerlyzers within the intervention arm. These may be seen as exclusive services rather than as shareable information. With this residual chance of contamination taken into account, the smoking cessation rate in the intervention arm was statistically significantly higher than in the control arm. Overall, smokers who received the intervention were almost three times more likely to stop smoking (aOR 2.92 95% confidence interval 1.56–5.50) compared to those who did not receive it.

The sample size was powerful enough for the effect size. Hence, the beneficial impact of the intervention service package within the current trial is generalizable. This rate of stopping smoking is higher than those reported in previous studies carried out in Thailand where there is lack of primary health care-based smoking cessation trial [24].

Strong evidences showed that smoking-cessation intervention are cost-effective even with resource-intensive intervention because of quality-adjusted life years (QALYs) gained due to better health after giving up smoking [25]. ESCAPE study utilized intervention components which are locally available resources. Primary health care nurses and family members are human resources required to start and sustain the intervention. Smoking cessation diary can be printed in any local context. NRT applied in ESCAPE intervention were local-made generic nicotine replacement chewing gum which cost less than 2–3 US dollar for 10 pieces-strip. It was an optional NRT, thus, just a few percentage of smokers used it in this study. Additionally, piCO+ Smokerlyzer is the only equipment new to the local health service. One smokerlyzer can be used for years. Overall, intervention components are simple and feasible in the real world primary health care setting of Thailand. Hence, we expect that evidence from its finding are ready for translation into policy and practice, to be scaled up in the resource-limited setting of LMICs.

Thailand is well known for its distinctive tobacco control model. Series of national anti-tobacco legislations, taxation on tobacco products, pictorial warning on cigarettes packages, smoke-free public spaces, health protection rules and health promotion activities are well complying with World Health Organization Framework Convention on Tobacco Control (WHO FCTC) [26]. Recent literature within Thailand pointed out that hand-rolled cigarettes or roll-your own cigarettes and home-grown tobacco are the remaining challenges leading to abundant use of local tobacco product in rural areas and the need for smoking cessation services in the communities [26]. The effectiveness reported in this intervention this study may help to fill up the need for such smoking cessation services in the primary health care and communities of Thailand, leading towards a model country with the universal access to smoking cessation.

## Conclusion

While tobacco-smoking is globally regarded as the major cause of death from cancer, cardiovascular diseases, and many other diseases, smoking cessation has often been overlooked in developing countries where the burden of tobacco-related diseases such as CVD and cancer are progressively high [27]. This situation calls for a smoking cessation intervention which has been practically trialed and proven to be effective [18]. The intervention designed and tested in this ESCAPE trial has shown effective results within the primary health care setting of northern Thailand which may be replicated in similar developing country contexts around the world.
